# Identifying ambulatory care sensitive conditions: a systematic review of studies defining sets of diseases with avoidable hospitalisations in European countries

**DOI:** 10.1136/bmjopen-2025-112777

**Published:** 2026-03-26

**Authors:** Daniel Martinho-Dias, Bernardo Sousa-Pinto, Mariana Canela-Pais, Ana Garrido-Oliveira, Dulce Pinto, Altamiro Costa-Pereira, Tiago Taveira-Gomes, João Almeida Fonseca, António José de Almeida Soares

**Affiliations:** 1 RISE-Health, Department of Community Medicine, Information and Health Decision Sciences, Faculty of Medicine, University of Porto, Porto, Portugal; 2 USF Ao Encontro da Saúde, Unidade Local de Saúde do Médio Ave, Santo Tirso, Portugal; 3 Unidade Local de Saúde do Alto Minho EPE, Viana do Castelo, Portugal

**Keywords:** Primary Care, Emergency Departments, Systematic Review

## Abstract

**Abstract:**

**Background:**

Ambulatory care sensitive conditions (ACSC) are health conditions that can be adequately managed in the outpatient setting. Timely treatment and interventions may avoid the need for hospitalisation and emergency department visits.

**Objectives:**

We aimed to identify ACSC lists developed for European populations.

**Design:**

Systematic review. We included primary studies that aimed to develop a list of ACSC for the general population or subpopulations within European countries. Studies reporting a formal methodology were eligible. Systematic or narrative reviews, and protocols were excluded.

**Data sources:**

PubMed, Web of Science and Scopus were searched on 21 October 2025. Data search was complemented with the search for ‘ambulatory OR preventable’ in the websites of the WHO Regional Office for Europe, OECD (Organisation for Economic Co-operation and Development) and NHSOF (British National Health Service Outcomes Framework).

**Data extraction and synthesis:**

Two reviewers independently collected data on type of population, geographical coverage, bibliographic support, use of qualitative or quantitative methods, ontology system, as well as the identified conditions per list. Data on methodological characteristics was qualitatively synthesised. Conditions identified as ACSC were aggregated under International Classification of Diseases 10th Revision (ICD-10). Each primary study with Delphi component was assessed using the Diamond *et al* risk of bias tool. Studies with a qualitative component were assessed using Joanna Briggs Institute (JBI) checklist for qualitative research.

**Results:**

A total of 12 articles were included. Six European countries have lists developed for general populations. A total of 263 unique ACSC have been defined (932 codes) for the general populations. For the paediatric age, 28 conditions (70 codes) were identified, while 37 diagnoses (58 codes) were listed for the nursing home population. Most commonly identified ACSCs were infection-related, chronic cardiovascular or respiratory diseases. Delphi methods were employed in eight studies, with a median of 3 (2–3.25) rounds with a median of 36.5 (32.8–42.5) panellists. Risk of bias assessment yielded a quality score of 2 (out of 4) for six studies and of 3 for the remaining two studies. Remaining studies were assessed with JBI yielding a median 6.5 (6.0–7.0) points (out of 10 possible points). The most used code system was ICD-10. Use of nationwide real-world databases was limited to six studies. No identified ACSC lists explicitly fulfilled all criteria defined by Solberg and Weissman for ACSC establishment.

**Conclusions:**

The evidence on ambulatory care sensitive conditions is heterogeneous and derives from different methodologies and covers six European countries. Most lists are diagnosis-based, aim at national, general populations and include Delphi components to define ACSC. We speculate that the future inclusion of primary care data could enhance ACSC evidence.

**PROSPERO registration number:**

CRD42022349270.

**STRENGTHS AND LIMITATIONS OF THIS STUDY**

- Comprehensive query of electronic databases and governmental data sources for the development of ambulatory care sensitive conditions lists for European countries.
- Includes a thorough characterisation of methodologies employed for list development.
- Population type and coverage per list are reported.
- Identified conditions have been aggregated under International Classification of Diseases 10th Revision.
- Lack of comparable quantitative data to synthesise is a limitation.

## Introduction

 Ambulatory care sensitive conditions (ACSC) can be defined as health conditions that can undergo adequate management in the outpatient setting if treatment and interventions are timely delivered, with the potential avoidance of the need for hospitalisation and emergency department visits.^1^

Hospitalisations for ACSC are currently used as an outcome indicator of access to primary care and its quality.^2^,^7^ Indeed, the use of ACSC as a performance indicator is currently endorsed by many international organisations.^1^ These include the WHO Regional Office for Europe,^8^ the Organisation for Economic Co-operation and Development (OECD),^9 10^ the British National Health Service Outcomes Framework (NHSOF)^11^ and the American Agency for Healthcare Research on its Quality Prevention Quality Indicators.^12 13^ The conditions deemed as ACSC are not uniform, and the published lists are mostly directly endorsed by governmental programmes.

Estimates of the economic relevance of ACSC hospitalisations vary across Europe, with different magnitudes according to used methods and the size of the health systems. ACSC-related hospital costs account for several billion euros annually in large health systems and hundreds of millions in smaller countries.^14^,^17^ Despite their relevance from the public health and economic standpoints, one of the earliest attempts to analyse avoidable hospitalisations started with Billings *et al*,^18^ who analysed a list of conditions they deemed as preventable. The authors concluded that patients with poor socioeconomic status and those uninsured or with basic coverage insurance were susceptible to increased hospitalisation rates for ambulatory sensitive conditions. The work of Billings *et al* followed that of Solberg *et al* and Weissman *et al*,^19 20^ who attempted to establish formal criteria for the definition of an ACSC. These criteria include (1) bibliographic support (ie, the existence of previous studies indicating that a certain condition may be an ACSC); (2) yearly hospitalisation rates of 1/10 000 population or higher; (3) clarity of condition definition and coding of diagnoses (4) potentially avoidable admission through primary care; and (5) hospitalisation is needed when the health problem occurs. Rooted in the development of these criteria is the idea that ACSC lists are not necessarily universally applicable either across countries nor across subpopulations with different characteristics. That is, what can be considered an ACSC depends on the country or population which is being considered. In addition, ACSC are consistently identified as relevant for primary care assessment, although specific lists have not been developed for all countries.

We need to better understand current evidence on ACSC, and how it has been generated and validated, particularly when used in primary care performance assessment or to inform health policy. To the best of our knowledge, evidence synthesis efforts on ACSC and on methodologies employed for the development of ACSC lists have not been made for European settings. In this study, we aim to systematically identify and characterise the methodological development of ACSC lists developed for populations in European countries.

## Methods

This systematic review was written in accordance with the PRISMA (Preferred Reporting Items for Systematic Review and Meta-Analysis)-2020 reporting guidelines.^21^

### Eligibility criteria

We included primary studies that aimed to develop an ACSC list for the general population or subpopulations within European countries. Only studies that reported the methodology used for the development of the ACSC list were considered. Systematic or narrative reviews, and protocols were excluded. There were no language, date or publication status restrictions.

### Information sources and search strategy

We searched three electronic bibliographic databases—PubMed, Web of Science and Scopus—on 21 October 2025. A search strategy was developed for each searched database. Terms were selected from primary studies on ACSC and from other systematic reviews.^1622^,^27^ The final search strategies can be consulted in online supplemental appendix 1. The electronic bibliographic search was complemented with the search for ‘ambulatory OR preventable’ in the websites of the WHO Regional Office for Europe, OECD and NHSOF. The references of included studies were checked for other studies that could have been missed from search strategy results. We manually searched for new ACSC lists within the studies identified during the screening phase that did not aim at list development per se. Those lists were to be included if the primary study developing them was found.

### Selection process

Search results were deduplicated and screened independently by two reviewers first by title and abstract and subsequently by full-text reading.^28^ Final deduplication was manual but supported by Rayyan's built-in duplicates finder feature. Screening was conducted within the Rayyan platform, but no decision automation tools were used. Conflicts between reviewers were solved by consensus. Authors were contacted whenever available information was not sufficient to allow a decision on the study inclusion. A 4-week window for reply was provided.

### Data collection process

Data was independently collected by two reviewers and introduced into a purposely-built Excel file. A pilot version of the form was initially developed and modified after testing. Collected data was merged into a single file and conflicts were resolved through consensus between the two reviewers. In the case of unclear records or definitions, authors were contacted for clarification and a 4-week window for reply was provided. There was no data imputation. Collected variables per study were listed as ACSC as well as variables related to study metadata, study design and characteristics of studied databases and Delphi panels. We also collected data on the presence of Solberg and Weissman criteria. The full set of variables is reported in online supplemental appendix 2. Studies in languages that the authors were not fluent in were translated to English using the DeepL system. We made translations from the Italian, German, Spanish and Finnish languages. We did not collect or treat individual patient data.

### Risk of bias assessment

All studies with a qualitative component were assessed using Joanna Briggs Institute (JBI) critical appraisal tool for qualitative studies. Most studies included a consensus process through the Delphi method. Quality assessment of the Delphi method was based on the following questions by Diamond *et al*^29^ ‘Were criteria for participants reproducible?’. ‘Was the number of rounds to be performed stated?’, ‘Were criteria for dropping items clear?’, ‘Stopping criteria other than rounds specified?’. Both risk of bias assessment and Delphi quality were independently conducted by two reviewers and conflicts were solved by consensus.

### Data analysis

Descriptive statistics are presented for each variable of interest (please address online supplemental appendix 2 for further detail). Studies characteristics, such as study designs, target population, setting, the number of conditions identified, their variation across lists and across subpopulations were tabulated. For each study, we assessed the five Solberg and Weissman criteria^19 20^ for ACSC establishment.

Identified conditions in each list were grouped in larger categories for improved information management. To create these categories, we identified all second-level parent International Classification of Diseases (ICD) codes and created new categories that would map for both ICD-9 and ICD-10 tables. Identified ACSC were then included under these new categories (with information on studies supporting them). A study without a code system^30^ has been manually attributed an ICD-10 code. Original codes were kept for traceability regarding condition grouping. No meta-analysis was conducted due to its inadequacy to answer the research question.

Regarding Delphi studies characterisation, we considered the panel members that completed all rounds and excluded pilot panels. If the consensus threshold was asymmetric between rounds, we considered the average threshold.

### Patient and public involvement

There was no involvement of patient or public during the elaboration of this systematic review.

## Results

### Study selection

**Figure 1:**
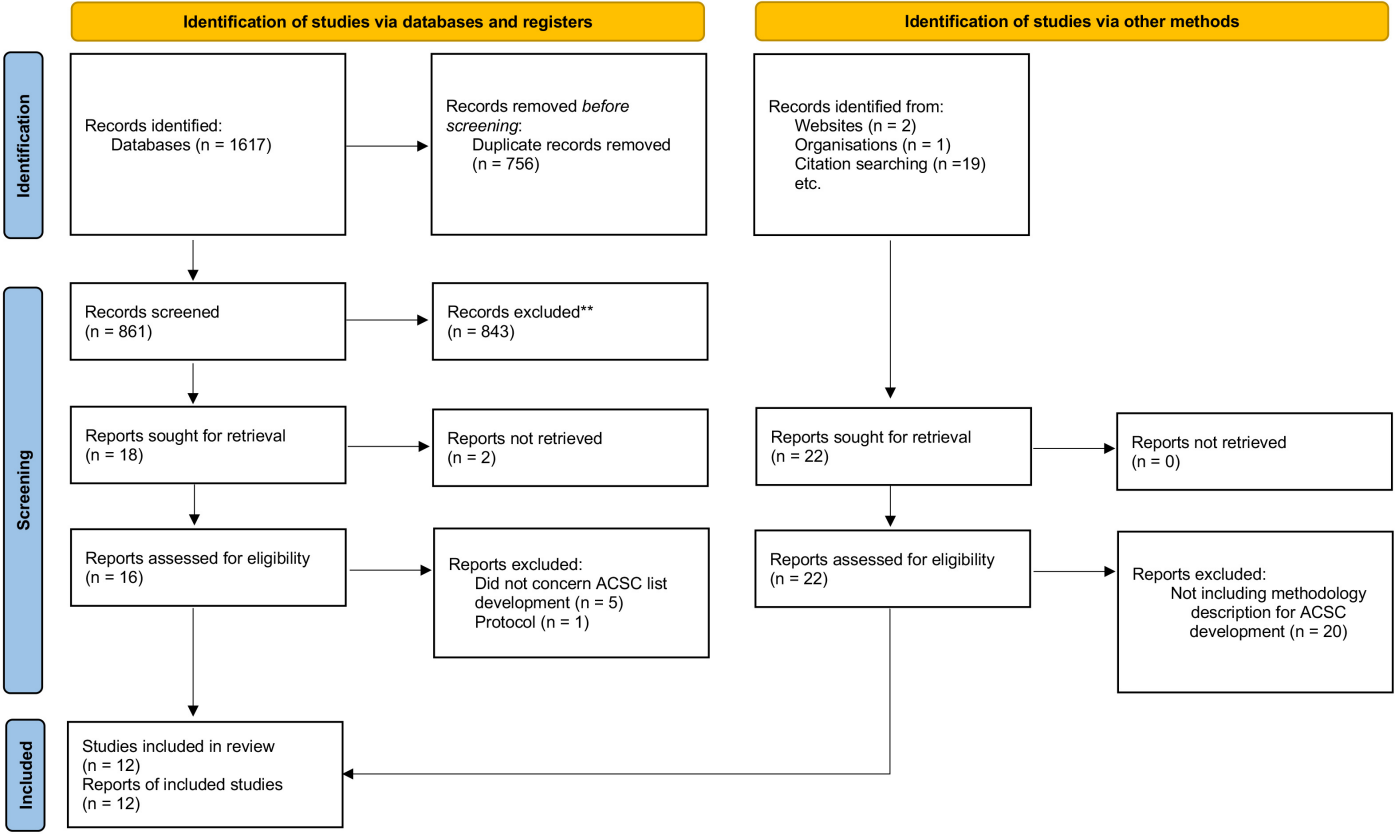
Preferred Reporting Items for Systematic Review and Meta-Analysis flow diagram on primary studies including the development of lists of ambulatory care sensitive conditions for Europe.^21^ All included countries are described in online supplemental appendix 1. ACSC, ambulatory care sensitive conditions.

Our electronic bibliographic search retrieved a total of 1617 articles, of which 756 deduplicated records were screened for inclusion. A total of 10 primary studies meeting eligibility criteria were included. Two extra articles were included from manual reference checking, on authors’ contacts for clarification or via health authorities’ webpages search. Therefore, a total of 12 included articles were included^1630^,^40^ (figure 1).

### Study characteristics

Study characteristics are summarised in table 1. Included ACSC lists had different target populations—nine lists developed for the general population of their target country/region,^1631^,^37 40^ two lists were developed for paediatric patients,^36 38^ one for nursing home residents^39^ and one for research prioritisation purposes.^30^ Most lists considered hospital admissions and/or discharge diagnoses for ACSC definition, while two considered emergency department episodes for defining ambulatory care.^31 37^ Germany,^31 37 39^ UK,^33^ Portugal,^16^ Latvia,^32^ Spain^35^ and Ukraine^40^ were the only European countries with developed ACSC lists for their general population with a national scope. In Italy, an ACSC list has been developed, but for the Bologna region.^36^

**Table 1:** Included studies and reported methodologies for ACSC list development

Target population	Authors	Main setting	Bibliographic support	Qualitative	Quantitative	A posteriori evaluation	Code system
General population							
Germany	Schuettig and Sundmacher^31^	ER-related ACSC	No	Yes (Delphi)	Yes. Nationwide insurance claims database	Yes. Evaluated the relationship between patient characteristics, ambulatory care settings, and the ER rate	ICD-10
Latvia	WHO European Regional Officer^32^	Admission-related ACSC	Yes. Based on a comprehensive search.	Yes (survey)	No	Frequency of patients hospitalised per condition; assessment of the frequency of readmissions	ICD-10
UK	Sanderson and Dixon^33^	Admission-related ACSC	No	Yes (Delphi)	Yes. Database of discharge diagnosis codes for the North West Thames Region	No	ICD-9
Portugal	Sarmento *et al*^16^	Admission-related ACSC	Yes. Based on a non-comprehensive search.	Yes (Delphi)	Yes. A frequency-based validation of Delphi results (national admissions database)	Two lists were developed. An extended list with Delphi’s ACSC+a core list with only frequency validated ACSC	ICD-10
Spain	Caminal *et al*^34^	Admission-related ACSC	Yes. Based on a non-comprehensive search.	Yes (Delphi)	Yes. Using a Catalonia admission database for the year 1996	No	ICD-9
Italy*	Pirani *et al*^36^	Admission-related ACSC	Yes. Based on a non-comprehensive search.	Yes (expert discussion without formal consensus panel)	No	Yes. Quantitative assessment of standardised rates of admissions in Emilia-Romagna Region	ICD-9
Spain	Caminal *et al*^35^	Admission-related ACSC	Yes. Based on a non-comprehensive search.	Yes (Delphi)	Yes. Database of hospital discharges obtained from the Catalan Health Service	Yes. Grouping of diagnosis codes and classification into type of intervention in ambulatory care	ICD-9
Germany	Sundmacher^37^	ER-related ACSC	Yes. Based on a comprehensive literature review	Yes (Delphi)	Yes. Database of all German hospital cases	Yes. A degree of preventability of selected ACSC and specified the medical actions and systemic changes	ICD-10 (ICD-9 mapped to ICD-10)
Ukraine	Lekhan ^40^	Admission-related ACSC	Yes. Based on a non-comprehensive search	Yes (survey)	Yes. Centralised databases of hospitalisations	Yes. Yearly preventable hospitalisation rates	ICD-10
Paediatric population							
UK	Jones *et al*^38^	Admission-related ACSC	No	Yes (advisory panel)	Yes. Database of inpatient data from Hospital Episode Statistics for all NHS hospitals	Yes. Evaluation of rate of admission variation for identified ACSC	ICD-10
Italy*	Pirani *et al*^36^	Admission-related ACSC	Yes. Based on a non-comprehensive search	Yes (expert discussion without formal consensus panel)	No	Yes. Quantitative assessment of standardised rates of admissions in Emilia-Romagna Region	ICD-9
Nursing home population							
Germany	Bohnet-Joschko (2022)^39^	Admission-related ACSC	No	Yes (Delphi)	Yes. Database of health insurance claims data	Yes. Condition frequency and costs’ evaluation	ICD-10
Research prioritisation							
UK	Purdy *et al*^30^	Admission-related ACSC	Yes. Based on a comprehensive literature review	Yes (Delphi)	Yes. Hospital discharge data (Hospital Episode Statistics) for south west area	Yes. Resource impact evaluation	ICD-10

* Regional scope; also includes paediatric population.

ACSC, ambulatory care sensitive conditions; ER, emergency room; ICD, International Classification of Diseases; N/A, not applicable; NHS, National Health Service.

Regarding Solberg and Weissman criteria: (1) a literature support was found in 8 lists,^1630 32 34^,^37 40^ but only three were based on a comprehensive literature search^30 32 37^ (2) 10 lists provided have clarity in definitions^1631^,^36 38^ (3) 5 lists had a relevance for public health criteria of 1/10 000 population or stricter^16 34 35 39 40^ (4) all lists made assessments on whether the diagnosis is potentially avoidable by timely and effective ambulatory care (5) 7 lists described that ACSC would be considered if there was a need for hospitalisation once the health problem produced.^33^,^3840^

Eight lists used a database of admissions, discharges or emergency room episodes. Although the nationwide scope of seven out of eight lists, three did not use a nationwide database, but rather a regional database.^33^,^35^

**Figure 2:**
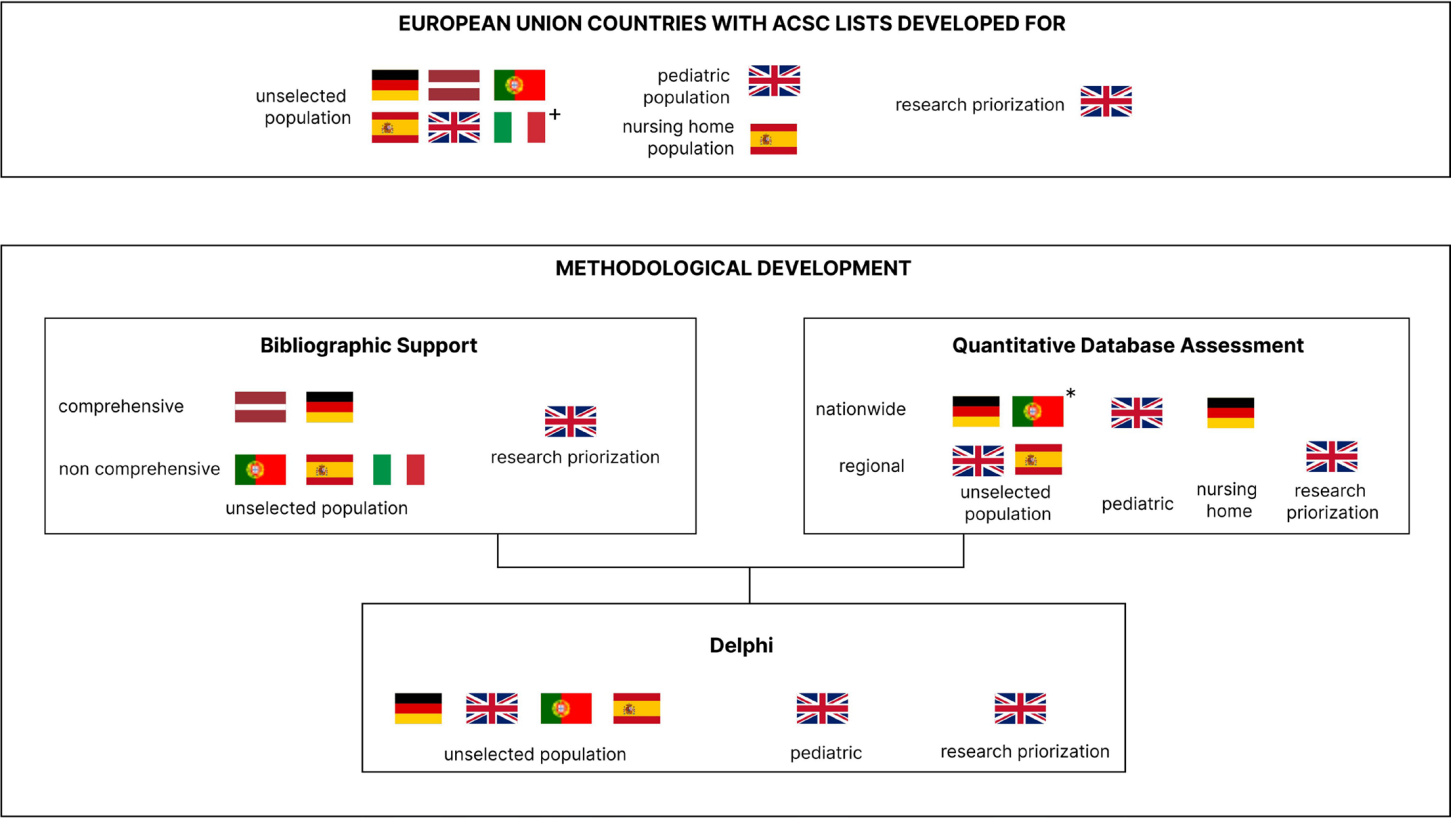
European countries in which list development has been conducted and respective methodological phases. +Regional population, *nationwide frequency validation post-consensus method. ACSC, ambulatory care sensitive conditions.

A Delphi method was employed in eight studies,^1630 31 33^,^35 37 39^ usually as the main driver of ACSC selection. Four lists did not employ formal Delphi consensus methods.^8 32 36 38 40^ A median (IQR) of 3.0 (2.0–3.25) Delphi rounds were conducted per study, with a median (IQR) threshold for consensus of 75 (70–75) % of panellists. The median number of panellists that completed all rounds was 36.5 (32.75–42.5). Three studies included a pre-round pilot.^30 31 34^ Figure 2 ensues the European countries in which list development has been conducted, along with the respective methodological phases.

All lists referred to ACSC by using coding systems. The most frequently used coding system was ICD-10 (eight lists)^1630^,^32 37^ followed by ICD-9.^33^,^36^

### Conditions identified as ambulatory care sensitive conditions

Studies were heterogeneous in number and degree of detail with which the identified conditions were reported.

Amidst all included studies, a total of 1137 unique codes corresponding to 347 unique conditions were identified as ACSC. Each list identified on average 94.8 (73.7) codes and 28.9 (16.7) conditions.

The smallest list^38^ had six identified conditions while the biggest listed 64.^37^

Considering only second-level ICD-10 or their ICD-9 equivalents, a total of 114 ACSC were identified. Box 1 and table 2 list these conditions if they are present in at least half of the included lists. Further conditions and different overviews are presented as supplementary material, which is accessible at https://marianacpais.github.io/ambulatory_tables/.

Box 1 **Identified ambulatory care sensitive conditions (ACSC) present across 50% or more of included primary studies (groupings of conditions). The full list of conditions and origins is available at ambulatorycare.med.up.pt.**

**Multicountry ACSC**

Full list of conditions available at: ambulatorycare.med.up.pt.

- Anaemias.
- Chronic lower respiratory diseases.
- Diabetes mellitus.
- Diseases of oesophagus, stomach and duodenum.
- Episodic and paroxysmal disorders.
- General symptoms and signs.
- Hypertensive diseases.
- Infections of the skin and subcutaneous tissue.
- Inflammatory diseases of female pelvic organs.
- Influenza and pneumonia.
- Intestinal infectious diseases.
- Ischaemic heart diseases.
- Metabolic disorders.
- Non-infective enteritis and colitis.
- Renal tubulo-interstitial diseases.
- Symptoms and signs involving the circulatory and respiratory systems.
- Tuberculosis.
- Viral and prion infections of the central nervous system.
- Viral infections characterised by skin and mucous membrane lesions.

**Table 2:** List of codes from primary studies reporting to parent categories common across at least two thirds of the articles

Parent category	Reported ICD-9 codes	Reported ICD-10 codes
Other bacterial diseases	032, 033, 037	A33, A34, A35, A36, A37, A46
Diabetes mellitus	250, 251, 250.1, 250.2, 250.3, 250.4, 250.5, 250.6, 250.7, 250.8, 250.9	E10, E10.0, E10.1, E10.2, E10.3, E10.4, E10.5, E10.6, E10.7, E10.8, E10.9, E11, E11.0, E11.1, E11.2, E11.3, E11.4, E11.5, E11.6, E11.7, E11.8, E11.9, E12, E12.0, E12.1, E12.2, E12.3, E12.4, E12.5, E12.6, E12.7, E12.8, E12.9, E13, E13.0, E13.1, E13.2, E13.3, E13.4, E13.5, E13.6, E13.7, E13.8, E13.9, E14, E14.0, E14.1, E14.2, E14.3, E14.4, E14.5, E14.6, E14.7, E14.8, E14.9
Chronic lower respiratory diseases	490, 491, 492, 493, 494, 496, 493.9	J40, J41, J42, J43, J44, J45, J46, J47
Infections of the skin and subcutaneous tissue	681, 682, 683, 686	L00, L01, L02, L03, L03.0, L04, L05, L08, L08.0, L08.8, L08.9
Renal tubulo-interstitial diseases	590, 599, 590.1, 590.2, 590.3, 590.4, 590.5, 590.6, 590.7, 590.8, 590.9	N10, N11, N12, N13.6
Hypertensive diseases	401, 402, 403, 404, 405, 402.01, 402.1, 402.11, 402.91	I10, I10.0, I10.9, I11, I11.0, I11.9, I12, I13, I13.0, I13.1, I13.2, I13.9, I15
Other forms of heart disease	428	I48, I49.8, I49.9, I50
Diseases of oesophagus, stomach and duodenum	531, 532, 533, 531.2, 531.4, 531.6, 531.9, 532.2, 532.4, 532.6, 533.2, 533.4, 533.6, 535.5, 536.8	K21, K25, K25.0, K25.1, K25.2, K25.4, K25.5, K25.6, K25.9, K26, K26.0, K26.1, K26.2, K26.4, K26.5, K26.6, K27, K27.0, K27.1, K27.2, K27.4, K27.5, K27.6, K28.0, K28.1, K28.2, K28.4, K28.5, K28.6, K29, K29.7, K29.9, K30, K31
Influenza and pneumonia	481, 483, 485, 486, 482.2, 482.3, 482.9	J09, J10, J11, J12, J13, J14, J15, J15.3, J15.4, J15.7, J15.8, J15.9, J16, J16.0, J16.8, J18, J18.0, J18.1, J18.8, J18.9

ICD, International Classification of Diseases.

No conditions were common to all lists; 37 (31.2%) of conditions were present in at least one-third of developed lists, with 9 (7.8%) present in over two-thirds of all lists.

Regarding general populations only, a total of 263 unique ACSC have been defined (932 codes). For the paediatric age, 28 conditions (70 codes) were identified,^36 38^ and 37 diagnoses (58 codes) for the nursing homes population.^38^ A research prioritisation exercise for the UK identified 18 unique codes (10 conditions).^38^

The identified ACSC are summarised in supplementary table S2 (http://ambulatorycare.med.up.pt/) according to their closest general term, as lists varied in the degree of specificity. Most commonly identified ACSC (in at least nine lists) were cardiovascular or with cardiovascular complications (‘Diabetes mellitus’, ‘Hypertensive diseases’, ‘Other forms of heart disease’), chronic respiratory (‘Chronic lower respiratory diseases’) and infectious (‘Influenza and pneumonia’, ‘Infections of the skin and subcutaneous tissue’, ‘Other bacterial diseases’).

### Risk of bias in studies

No ACSC list identified in the primary studies explicitly fulfilled all five criteria defined by Solberg et al^38^ and Weissman *et al*,^19 20^ but ten lists fulfilled at least 3/5 criteria. Most studies provided clarity in the definition of conditions (11/12),^1632^,^38 40^ defined ACSC as potentially avoidable by timely and effective ambulatory care (10/12)^1631 33^,^38 40^ and were supported on previous literature (9/12).^1630^,^32 34^ Seven studies explicitly mentioned defining ACSC regarding the necessity of hospitalisation once the health problem occurs.^33^,^3840^ Only four studies required a condition to be relevant for public health (a yearly hospitalisation rate of at least 1 per 10 000 population) to define it as a potential ACSC.^1634^,^36 39^

Application of the JBI critical appraisal tool for qualitative results yielded a median 6.5 (6.0–7.0) points out of 10 possible points. No studies reported on question 6 (regarding locating the research culturally), on question 7 (regarding the influence of the researcher on the research, and vice versa), nor on question 8 (related to patient involvement) of the appraisal tool. All studies were consistent regarding question 4 (on congruity between the research methodology and data analysis), and question 10 (conclusions drawn from analysis).

**Figure 3:**
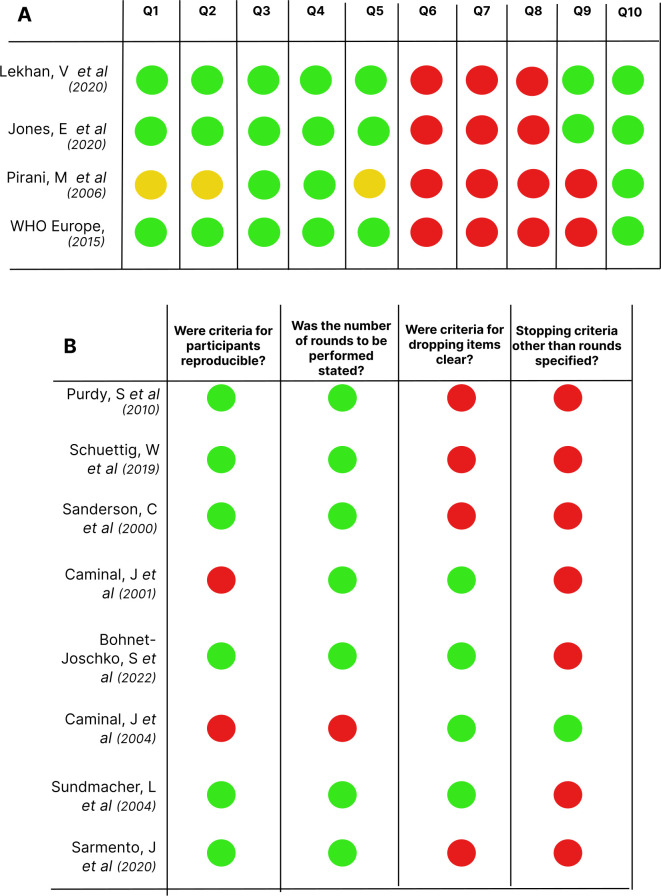
(A) Risk of bias assessment using Joanna Briggs Institute critical appraisal tool for qualitative studies. Green— yes; yellow—unclear; red—no. (B) Quality of qualitative reporting for each included study with a Delphi component (n=8), according to Diamond *et al* items.^29^ Green—risk of bias not present; red—risk of bias present or no information available regarding item.

Eight studies including Delphi methods were evaluated according to Diamond *et al* tool for quality of methodological reporting and were attributed a quality score.^29^ Regarding the final quality score, six studies achieved two points^1630 31 33^,^35^ and two studies received three points^37 39^ (out of four possible points) according to Diamond *et al* criteria.^29^ The item most frequently classified as ‘low risk of bias’ was that on the statement of the number of rounds (7/8).^16 30 31 33 34 37^ Reproducibility of participant criteria for panels was present in six studies,^16 30 31 33 37 39^ while four stated criteria for dropping items.^34 35 37 39^ Only one article specified stopping criteria other than rounds.^37^ The risk of bias assessment as well as the reporting quality for Delphi studies is summarised in figure 3.

## Discussion

### Findings

In this study, we systematically identified available ACSC for European populations and methodologically characterised them. We identified lists covering six European countries (Germany, Latvia, the UK, Portugal, Spain, Ukraine). We learnt that most primary studies included lists which are diagnosis-based, aim at national, general populations and include Delphi components to define ACSC. The most consensual conditions across lists were infectious diseases, chronic cardiovascular (diabetes, hypertension) and chronic respiratory diseases. However, eligible primary studies did not follow all desirable methodological steps for list development. Previous bibliographic support, as defined by Solberg and Weissman as relevant criteria for ACSC development, is not present in all studies: only one-third report conducting a comprehensive literature search prior to ACSC list development, with another third not reporting any search prior to qualitative or quantitative study implementations. Support from real-world evidence databases is limited as most studies did not include a nationwide database or a representative sample of nationwide cases. Often, consensus panels attempt to establish ACSC for all countries and their general populations^1631 33^,^35 37^ having the support of regional databases or insurance claim databases. We did not find the use of any database including primary care data, which we believe could prove of benefit to establish referrals processes and the health user pathway within the healthcare system. We also found some relevant limitations in the employed methodologies for achieving consensus (figure 2). List development frequently relies on Delphi assessments. The Delphi method is an iterative, multiround process designed to achieve expert consensus and is widely applied in guideline development and priority setting. While it is valuable in contexts where empirical evidence is limited, the methodology is subject to limitations, including variability in consensus definitions and risks of selection bias and unrepresentative expert input.^2641^,^44^ Despite this, almost all Delphi components included over 20 experts, which is in line with a previous recommendation that the panel must include at least 20 experts to provide robust estimates in a Delphi exercise.^45^ Overall, we consider the available evidence on list development to be mostly qualitative, in low quantity and of low-medium quality.

### Strengths and limitations

To the best of our knowledge, this is the first systematic review on the topic to include a methodological characterisation of developed lists and to focus on the European setting. We believe our eligibility criteria to be clear and appropriate to answer our question, and our search was comprehensive. We have identified available primary evidence, its quantity, discrepancy in methods and its potential flaws, informing on risk of bias for qualitative components and measuring the application of Solberg^19^ and Weissman criteria.^20^ We consider the aggregation of conditions under the ICD coding system to be useful for clearer definitions. We have created a webpage for easier data access and increased readability https://ambulatorycare.med.up.pt.

We also have important limitations. The evidence supporting ACSC list development is limited and methodologically heterogeneous. Few studies report comprehensive literature reviews or the use of nationally representative data, and none incorporate primary care databases. Furthermore, we also did not have access to individual patient data or to explore publication bias.

### Comparison with previous literature

The literature on application of previously developed lists for admission rates and temporal trends of diagnosis^46^,^49^ is larger than literature reporting the development of ACSC reviews, which have focused on primary care accessibility,^50^ organisational aspects^22^ and continuity of ambulatory care.^51^ Previous syntheses of evidence have also been focused on specific populations, such as the paediatric patients,^52 53^ migrants^54^ and those in need of palliative care.^55^ Interventions aimed at reducing ACSC hospitalisations have also been evaluated,^24^ as well as costs.^56^ None of these reviews address the topic of methodological characterisation and assessment, nor do they focus on European population.

### Meaning of the study

This work has some relevant implications. The evidence for European countries has methodological fragilities, with insufficient external validity and generalisability. This calls for caution when interpreting literature on temporal or regional trends in ACSC. It also calls for caution when employing ACSC-related hospitalisations to assess primary care and establish pay-for-performance models. Although the evaluation of this relationship is commonly used in diverse countries,^2^,^711 57^ we believe these assessments would largely benefit from stronger evidence to yield fairer evaluations and to safely improve ambulatory care. We also found that all developed lists are diagnosis-based. While this may be extremely relevant from the public health perspective, their relevance for the referring physician in the primary care setting may be less relevant. The primary care clinicians are most likely the most relevant stakeholders in determining whether the patient will be treated in the ambulatory care setting or referred. Their decision is frequently based on clinical presentation rather than raw diagnosis, and in their ability to timely access diagnostic tools that safely exclude the need for urgent care in doubtful cases.^58^,^60^

### Unanswered questions and future research

We have mapped available lists for European countries, but quantitative validation from individual patient data is missing and some methodological fragilities identified by risk of bias tools should be addressed in future studies. It also remains unclear which are the variables that influence consensus, and the reasons for the large variations in lists observed across countries. We believe some effort should be put towards the creation of a common framework for the development of validated lists per population.

From a direct public health perspective, future research should be directed into improving ACSC condition selection and definition, including both clinical and user perspectives in the selection of conditions and in the identification of barriers to ambulatory care.

From a primary care standpoint, we consider that future research should focus on generating and aggregating evidence for primary care referral. This could reduce clinical uncertainty at the ambulatory settings. Improved decision-making at the referral decision time could then lead to a safer and more efficient shift of certain patients from the hospital to the ambulatory care setting.

## Conclusion

Despite the public health and economic relevance of ACSC, there is a low volume of lists defining these conditions in Europe, with low agreement between lists and diverse methodologies employed for list development, mostly consisting of qualitative or mixed methods designs, with non-negligible risk of bias. There is a need for more robust evidence regarding ambulatory care sensitive conditions. Such data would better inform policymakers, thereby facilitating future policies designed to safely streamline patient journeys within the healthcare system.

## Supplementary material

10.1136/bmjopen-2025-112777 online supplemental file 1

## Data Availability

Data are available in a public, open access repository.
