# Supplementary material for: Identifying ambulatory care sensitive conditions: a systematic review of studies defining sets of diseases with avoidable hospitalisations in European countries
Source: BMJ Open. 2026 Mar 26;16(3):e112777. doi: 10.1136/bmjopen-2025-112777 (PMC13034391; doi:10.1136/bmjopen-2025-112777)
Supplement: online supplemental file 1 [file bmjopen-16-3-s001.docx]

**Appendix 1:** Search strategy developed per database. Databases were queried on 10 June 2022.

| **Pubmed** |
| --- |
| (“ambulatory care sensitive condition”[Title/Abstract] OR “ambulatory care sensitive conditions”[Title/Abstract] OR “ambulatory care sensitive”[Title/Abstract] OR “primary care sensitive”[Title/Abstract] OR “avoidable hospitalization”[Title/Abstract] OR “avoidable hospitalization”[Title/Abstract] OR “preventable hospitalizations”[Title/Abstract] OR “preventable hospitalisations”[Title/Abstract] OR “ACS condition*”[Title/Abstract] OR “ACS hospitali*”[Title/Abstract] OR “ACSCs”[Title/Abstract] OR “ACSH”[Title/Abstract] OR “ACSHs”[Title/Abstract] OR “ASSC”[Title/Abstract] OR “ASSCs”[Title/Abstract] OR “H-ACSC”[Title/Abstract] OR “H-ACSCs”[Title/Abstract] OR “AH-ACSC”[Title/Abstract] OR “AH-ACSCs”[Title/Abstract] OR “PHCSC”[Title/Abstract] OR “PHCSCs”[Title/Abstract] OR “unplanned hospital admission”[Title/Abstract] OR “care sensitive”[Title/Abstract] OR “preventable admission”[Title/Abstract] OR “avoidable admission”[Title/Abstract])  AND  ((Austria[Title/Abstract] OR Austrian[Title/Abstract]) OR (Belgium[Title/Abstract] OR Belgian[Title/Abstract]) OR (Bulgaria[Title/Abstract] OR Bulgarian[Title/Abstract]) OR (Croatia[Title/Abstract] OR Croatian[Title/Abstract]) OR (Czech Republic[Title/Abstract] OR Czech[Title/Abstract]) OR (Denmark[Title/Abstract] OR Danish[Title/Abstract]) OR (England[Title/Abstract] OR English[Title/Abstract]) OR (Finland[Title/Abstract] OR Finnish[Title/Abstract]) OR (France[Title/Abstract] OR French[Title/Abstract]) OR (Germany[Title/Abstract] OR German[Title/Abstract]) OR (Greece[Title/Abstract] OR Greek[Title/Abstract]) OR (Hungary[Title/Abstract] OR Hungarian[Title/Abstract]) OR (Italy[Title/Abstract] OR Italian[Title/Abstract]) OR (Latvia[Title/Abstract] OR Latvian[Title/Abstract]) OR (Lithuania[Title/Abstract] OR Lithuanian[Title/Abstract]) OR (Malta[Title/Abstract] OR Maltese[Title/Abstract]) OR (Netherlands[Title/Abstract] OR Dutch[Title/Abstract]) OR (Poland[Title/Abstract] OR Polish[Title/Abstract]) OR (Portugal[Title/Abstract] OR Portuguese[Title/Abstract]) OR (Romania[Title/Abstract] OR Romanian[Title/Abstract]) OR (Scotland[Title/Abstract] OR Scottish[Title/Abstract]) OR (Slovakia[Title/Abstract] OR Slovak[Title/Abstract]) OR (Spain[Title/Abstract] OR Spanish[Title/Abstract]) OR (Sweden[Title/Abstract] OR Swedish[Title/Abstract]) OR (United Kingdom[Title/Abstract] OR British[Title/Abstract]) OR (Wales[Title/Abstract] OR Welsh[Title/Abstract]) OR (Luxembourg[Title/Abstract] OR Luxembourgish[Title/Abstract]) OR (Slovenia[Title/Abstract] OR Slovenian[Title/Abstract]) OR (Cyprus[Title/Abstract] OR Cypriot[Title/Abstract]) OR (Estonia[Title/Abstract] OR Estonian[Title/Abstract]) OR (Ireland[Title/Abstract] OR Irish[Title/Abstract]) OR (Albania[Title/Abstract] OR Albanian[Title/Abstract]) OR (Armenia[Title/Abstract] OR Armenian[Title/Abstract]) OR Andorra[Title/Abstract] OR Andorran[Title/Abstract]) OR (Azerbaijani[Title/Abstract] OR Azerbaijani[Title/Abstract] OR Azeri[Title/Abstract]) OR (Belarus[Title/Abstract] OR Belarusian[Title/Abstract]) OR (Bosnia[Title/Abstract] OR Bosnian[Title/Abstract]) OR (Georgia[Title/Abstract] OR Georgian[Title/Abstract]) OR (Kazakhstan[Title/Abstract] OR Kazakhstani[Title/Abstract]) OR (Monaco [Title/Abstract] OR Monégasque[Title/Abstract]) OR (Montenegro[Title/Abstract] OR Montenegrin[Title/Abstract]) OR (Macedonia[Title/Abstract] OR Macedonian[Title/Abstract]) OR (Moldova[Title/Abstract] OR Moldovan[Title/Abstract]) OR (Russia[Title/Abstract] OR Russian[Title/Abstract]) OR (Serbia[Title/Abstract] OR Serb[Title/Abstract] OR Serbian[Title/Abstract]) OR (Ukraine[Title/Abstract] OR Ukrainian[Title/Abstract]) OR (Iceland[Title/Abstract] OR Icelandic[Title/Abstract] OR Icelander[Title/Abstract]) OR (Norway[Title/Abstract] OR Norwegian[Title/Abstract]) OR (Switzerland[Title/Abstract] OR Swiss[Title/Abstract]) OR (Turkey[Title/Abstract] OR Turkish[Title/Abstract]) OR Vatican[Title/Abstract] OR (Europe[Title/Abstract] OR European[Title/Abstract])) |
| **Web of Science** |
| (TS=”ambulatory care sensitive condition” OR TS=”ambulatory care sensitive conditions” OR TS=”ambulatory care sensitive” OR TS=”primary care sensitive” OR TS=”avoidable hospitalization” OR TS=”avoidable hospitalization” OR TS=”preventable hospitalizations” OR TS=”preventable hospitalisations” OR TS=”ACS condition*” OR TS=”ACS hospitali*” OR TS=”ACSCs” OR TS=”ACSH” OR TS=”ACSHs” OR TS=”ASSC” OR TS=”ASSCs” OR TS=”H-ACSC” OR TS=”H-ACSCs” OR TS=”AH-ACSC” OR TS=”AH-ACSCs” OR TS=”PHCSC” OR TS=”PHCSCs” OR TS=”unplanned hospital admission” OR TS=”care sensitive” OR TS=”preventable admission” OR TS=”avoidable admission”)    AND  (TS=Austria OR TS=Austrian OR TS=Belgium OR TS=Belgian OR TS=Bulgaria OR TS=Bulgarian OR TS=Croatia OR TS=Croatian OR TS=Czech Republic OR TS=Czech OR TS=Denmark OR TS=Danish OR TS=England OR TS=English OR TS=Finland OR TS=Finnish OR TS=France OR TS=French OR TS=Germany OR TS=German OR TS=Greece OR TS=Greek OR TS=Hungary OR TS=Hungarian OR TS=Italy OR TS=Italian OR TS=Latvia OR TS=Latvian OR TS=Lithuania OR TS=Lithuanian OR TS=Malta OR TS=Maltese OR TS=Netherlands OR TS=Dutch OR TS=Poland OR TS=Polish OR TS=Portugal OR TS=Portuguese OR TS=Romania OR TS=Romanian OR TS=Scotland OR TS=Scottish OR TS=Slovakia OR TS=Slovak OR TS=Spain OR TS=Spanish OR TS=Sweden OR TS=Swedish OR TS=United Kingdom OR TS=British OR TS=Wales OR TS=Welsh OR TS=Luxembourg OR TS=Luxembourgish OR TS=Slovenia OR TS=Slovenian OR TS=Cyprus OR TS=Cypriot OR TS=Estonia OR TS=Estonian OR TS=Ireland OR TS=Irish OR TS=Albania OR TS=Albanian OR TS=Armenia OR TS=Armenian OR TS=Andorra OR TS=Andorran OR TS=Azerbaijan OR TS=Azerbaijani OR TS=Azeri OR TS=Belarus OR TS=Belarusian OR TS=Bosnia OR TS=Bosnian OR TS=Georgia OR TS=Georgian OR TS= Kazakhstan or TS= Kazakhstani OR TS=Monaco OR TS= Monégasque OR TS=Montenegro OR TS=Montenegrin OR TS=Macedonia OR TS=Macedonian OR TS=Moldova OR TS=Moldovan OR TS=Russia OR TS=Russian OR TS=Serbia OR TS=Serb OR TS=Ukraine OR TS=Ukrainian OR TS=Iceland OR TS=Icelandic OR TS=Norway OR TS=Norwegian OR TS=Switzerland OR TS=Swiss OR TS=Turkey OR TS=Turkish OR TS=Vatican OR TS=Europe OR TS=European) |
| **Scopus** |
| (TITLE-ABS-KEY(“ambulatory care sensitive condition”) OR TITLE-ABS-KEY(“ambulatory care sensitive conditions”) OR TITLE-ABS-KEY(“ambulatory care sensitive”) OR TITLE-ABS-KEY(“primary care sensitive”) OR TITLE-ABS-KEY(“avoidable hospitalization”) OR TITLE-ABS-KEY(“avoidable hospitalization”) OR TITLE-ABS-KEY(“preventable hospitalizations”) OR TITLE-ABS-KEY(“preventable hospitalisations”) OR TITLE-ABS-KEY(“ACS condition*”) OR TITLE-ABS-KEY(“ACS hospitali*”) OR TITLE-ABS-KEY(“ACSCs”) OR TITLE-ABS-KEY(“ACSH”) OR TITLE-ABS-KEY(“ACSHs”) OR TITLE-ABS-KEY(“ASSC”) OR TITLE-ABS-KEY(“ASSCs”) OR TITLE-ABS-KEY(“H-ACSC”) OR TITLE-ABS-KEY(“H-ACSCs”) OR TITLE-ABS-KEY(“AH-ACSC”) OR TITLE-ABS-KEY(“AH-ACSCs”) OR TITLE-ABS-KEY(“PHCSC”) OR TITLE-ABS-KEY(“PHCSCs”) OR TITLE-ABS-KEY(“unplanned hospital admission”) OR TITLE-ABS-KEY(“care sensitive”) OR TITLE-ABS-KEY(“preventable admission”) OR TITLE-ABS-KEY(“avoidable admission”))  AND  (TITLE-ABS-KEY(Austria) OR TITLE-ABS-KEY(Austrian) OR TITLE-ABS-KEY(Belgium) OR TITLE-ABS-KEY(Belgian) OR TITLE-ABS-KEY(Bulgaria) OR TITLE-ABS-KEY(Bulgarian) OR TITLE-ABS-KEY(Croatia) OR TITLE-ABS-KEY(Croatian) OR TITLE-ABS-KEY(Czech Republic) OR TITLE-ABS-KEY(Czech) OR TITLE-ABS-KEY(Denmark) OR TITLE-ABS-KEY(Danish) OR TITLE-ABS-KEY(England) OR TITLE-ABS-KEY(English) OR TITLE-ABS-KEY(Finland) OR TITLE-ABS-KEY(Finnish) OR TITLE-ABS-KEY(France) OR TITLE-ABS-KEY(French) OR TITLE-ABS-KEY(Germany) OR TITLE-ABS-KEY(German) OR TITLE-ABS-KEY(Greece) OR TITLE-ABS-KEY(Greek) OR TITLE-ABS-KEY(Hungary) OR TITLE-ABS-KEY(Hungarian) OR TITLE-ABS-KEY(Italy) OR TITLE-ABS-KEY(Italian) OR TITLE-ABS-KEY(Latvia) OR TITLE-ABS-KEY(Latvian) OR TITLE-ABS-KEY(Lithuania) OR TITLE-ABS-KEY(Lithuanian) OR TITLE-ABS-KEY(Malta) OR TITLE-ABS-KEY(Maltese) OR TITLE-ABS-KEY(Netherlands) OR TITLE-ABS-KEY(Dutch) OR TITLE-ABS-KEY(Poland) OR TITLE-ABS-KEY(Polish) OR TITLE-ABS-KEY(Portugal) OR TITLE-ABS-KEY(Portuguese) OR TITLE-ABS-KEY(Romania) OR TITLE-ABS-KEY(Romanian) OR TITLE-ABS-KEY(Scotland) OR TITLE-ABS-KEY(Scottish) OR TITLE-ABS-KEY(Slovakia) OR TITLE-ABS-KEY(Slovak) OR TITLE-ABS-KEY(Spain) OR TITLE-ABS-KEY(Spanish) OR TITLE-ABS-KEY(Sweden) OR TITLE-ABS-KEY(Swedish) OR TITLE-ABS-KEY(United Kingdom) OR TITLE-ABS-KEY(British) OR TITLE-ABS-KEY(Wales) OR TITLE-ABS-KEY(Welsh) OR TITLE-ABS-KEY(Luxembourg) OR TITLE-ABS-KEY(Luxembourgish) OR TITLE-ABS-KEY(Slovenia) OR TITLE-ABS-KEY(Slovenian) OR TITLE-ABS-KEY(Cyprus) OR TITLE-ABS-KEY(Cypriot) OR TITLE-ABS-KEY(Estonia) OR TITLE-ABS-KEY(Estonian) OR TITLE-ABS-KEY(Ireland) OR TITLE-ABS-KEY(Irish) OR TITLE-ABS-KEY(Albania) OR TITLE-ABS-KEY(Albanian) OR TITLE-ABS-KEY(Armenia) OR TITLE-ABS-KEY(Armenian) OR TITLE-ABS-KEY(Andorra) OR TITLE-ABS-KEY(Andorran) OR TITLE-ABS-KEY(Azerbaijan) OR TITLE-ABS-KEY(Azerbaijani) OR TITLE-ABS-KEY(Azeri) OR TITLE-ABS-KEY(Bielorrus) OR TITLE-ABS-KEY(Belarusian) OR TITLE-ABS-KEY(Bosnia) OR TITLE-ABS-KEY(Bosnian) OR TITLE-ABS-KEY(Georgia) OR TITLE-ABS-KEY(Georgian) OR TITLE-ABS-KEY(Kazakhstan) OR TITLE-ABS-KEY(Kazakhstani) OR TITLE-ABS-KEY(Monaco) OR TITLE-ABS-KEY(Monégasque) OR TITLE-ABS-KEY(Montenegro) OR TITLE-ABS-KEY(Montenegrin) OR TITLE-ABS-KEY(Macedonia) OR TITLE-ABS-KEY(Macedonian) OR TITLE-ABS-KEY(Moldova) OR TITLE-ABS-KEY(Moldovan) OR TITLE-ABS-KEY(Russia) OR TITLE-ABS-KEY(Russian) OR TITLE-ABS-KEY(Serbia) OR TITLE-ABS-KEY(Serb OR TITLE-ABS-KEY(Serbian) OR TITLE-ABS-KEY(Ukraine) OR TITLE-ABS-KEY(Ukrainian) OR TITLE-ABS-KEY(Iceland) OR TITLE-ABS-KEY(Icelandic OR TITLE-ABS-KEY(Icelander) OR TITLE-ABS-KEY(Norway) OR TITLE-ABS-KEY(Norwegian) OR TITLE-ABS-KEY(Switzerland) OR TITLE-ABS-KEY(Swiss) OR TITLE-ABS-KEY(Turkey) OR TITLE-ABS-KEY(Turkish) OR TITLE-ABS-KEY(Vatican) OR TITLE-ABS-KEY(Europe) OR TITLE-ABS-KEY(European)) |

**Appendix 2:** Collected variables per study, when applicable.

| Identified conditions (and respective codes)  Study metadata   - Publication year - Authorship - Journal - Country   Study design characteristics:   - List scope (national *versus* regional) - Defined outcome (emergency room episodes *versus* admissions) - Bibliographic support and comprehensiveness of search methods - Presence of a quantitative component - Presence of a qualitative component - Application of Solberg and Weissman ACSC criteria   Studied database(s) – applicable to studies including a quantitative component   - Type - Geographical scope - Number of patients included - Coding system used (if any)   Delphi methods - applicable to studies including Delphi component   - Pilot study - Number of rounds - Number of members per round - Cut off levels for reaching consensus - Types of panelists included - Risk of Bias assessment: - Were criteria for participants reproducible? - Was the number of rounds to be performed stated? - Were criteria for dropping items clear? - Stopping criteria other than round specified?   Other methods employed for ACSC list development  Other *a posteriori* evaluation (e.g., ACSC frequency, costs per ACSC) |
| --- |
