# Supplementary material for: Identifying ambulatory care sensitive conditions: a systematic review of studies defining sets of diseases with avoidable hospitalisations in European countries
Source: BMJ Open. 2026 Mar 26;16(3):e112777. doi: 10.1136/bmjopen-2025-112777 (PMC13034391; doi:10.1136/bmjopen-2025-112777)
Supplement: Supplementary file 2 [file bmjopen-2025-112777.reviewer_comments.pdf]

PEER REVIEW HISTORY

BMJ Open publishes all reviews undertaken for accepted manuscripts. Reviewers are asked to complete a checklist review form and are provided with free text boxes to elaborate on their assessment. These free text comments are reproduced below.

ARTICLE DETAILS

Title (Provisional)

Identifying Ambulatory Care Sensitive Conditions: a systematic review of studies defining sets of diseases with avoidable hospitalisations in European countries

Authors

Martinho-Dias, Daniel; Sousa-Pinto, Bernardo; Canela-Pais, Mariana; Garrido-Oliveira, Ana; Pinto, Dulce; Costa-Pereira, Altamiro; Taveira-Gomes, Tiago; Fonseca, João A.; Soares, António José de Almeida

---

VERSION 1 - REVIEW

**Reviewer**	**1**
**Name**	**Kokkinakis, Ioannis**
**Affiliation**	**University of Lausanne**
**Date**	**11-Nov-2025**
**COI**	**None**

Thank you for the new revised version of the manuscript. Although the research question is interesting and important, the manuscript has many systematic and structural errors that need to be adressed (references in the text, stucture of the manuscript, syntax). Almost all parts (introduction, results, discussion, table 2...) need to be edited by the authors in order for the reader to understand the content. Table 2 could be more systematic and better structured as well as the text in the main manuscript. Data on Prospero are outdated and do not reflect the actual stage of the work. This work should be published, but only after a thorough resturcturing and correrctions from the authors.

**Reviewer**	**2**
**Name**	**Czajkowski, Marek**
**Affiliation**	**Örebro University, School of Medical Sciences**
**Date**	**13-Nov-2025**
**COI**	**None**

Thank you for the opportunity to peer-review this interesting study.

General Comments

This is a rather short publication (6 pages in current formatting, including a 1-page abstract) reporting an ambitious systematic review of the methods and the lists of Ambulatory Care Sensitive Conditions (ACSCs) for European countries. Due to divided focus the narrative is somehow difficult to follow. This is proved by quite ambiguous eligibility criteria: "primary studies that aimed to develop a list of ACSCs for the general population or subpopulations within European countries, and that used a properly described formal methodology." Both aims are interesting, but the synthesis of ACSC lists is a rather unique outcome and naturally deserves to be the main focus of the study. The methods of the included studies can be described and assessed as part of the systematic review process. Whatsmore, despite eligibility criteria requiring "a properly described formal methodology," studies reporting ACSC lists without such methodology were included (see Results, P6 L58), that needs adequate explanation. The outcomes of interest in primary studies are missing from the main text and are only listed in Appendix 2 as Collected variables per study, but unclear if pre-defined. Consequently, the PRISMA checklist has many "Not applicable" answers in the results section, where respective points should be reported for a systematic review.

Additional Comments

General

• The formatting of references should be more consistent: references should appear at the end of sentences, not at the beginning of a new one. For example, correct page 4 line 18 ". (14-17).", line 37 ". (22)", line 54 ".(26)", and page 5 line 18 ". (33)" etc.

• The use of ACSC in singular and plural (e.g., ACSC/ACSCs lists, "ACSC are …") and redundancies such as "ACSC conditions" (P6 L52) should be unified.

• The term "unselected population" is misleading throughout the article; consider using "general population" instead, as used in the study characteristics and the discussion.

Abstract

• In general, it is quite long. Consider removing some details not included in "PRISMA 2020 for Abstracts Checklist " or rewriting into shorter sentences.

• P3 L36: Correct spelling to "real-world".

• P3 L38: Regarding conclusions: "Evidence on ambulatory care sensitive conditions is heterogeneous and scarce, with considerable risk of bias." — it is unclear if evidence is truly scarce when 12 studies were found. Risk of bias was not assessed for all studies and is always considerable; please consider being more precise.

• P3 L36-38: "Most primary studies included lists which are diagnosis-based, aim at national, unselected populations, and include Delphi components to define ACSCs. This may limit

their usefulness to referring clinicians." — Please provide more details what is meant by "usefulness to referring clinicians" or remove the sentence from the abstract.

- P3 L38-39: "Future inclusion of primary care data could be of use to enhance ACSCs lists' usefulness." — Please clarify the origin of this statement, as it was stated that "Use of nationwide real-world databases was limited to six studies." meaning primary care data was included.

Introduction

- P4 L19: Remove "(s)" mid sentence and change the color of references (14-17) to black.
- P4 L20: Clarify the meaning of "public" in "Despite their public and economic relevance."
- P4 L21: Rewrite into shorter sentences and clarify what "they" refers to in "Despite their public and economic relevance, the earliest attempts to analyze avoidable hospitalizations started with Billings et al (18), who analyzed a list of conditions they deemed preventable and concluded that those patients with poor socioeconomic status and uninsured or with basic coverage insurance were susceptible to increased hospitalization rates for ambulatory sensitive conditions."
- P4 L29: Explain what is meant by "subpopulations" in "neither across countries nor across different subpopulations."
- P4 L31: The words "consistently" and "potentially" are somewhat contradictory in "ACSCs are consistently identified as potentially relevant for primary care assessment." Consider rephrasing.
- P4 L32: The statement regarding heterogeneity in methodological quality is unclear; provide a reference or move to the discussion.
- P4 L34-42: The discussion about the Delphi method and its limitations feels not appropriate for the introduction; move to the discussion.
- P4 L43: The speculation in "The potential absence of information regarding employed methodologies for list development or the further validation of lists used by health authorities for performance assessments yields reasonable questions regarding the usefulness of performance indicators built upon these lists for primary care evaluation" is not suitable for the introduction.
- P4 L46: The sentence "assess the employed methodologies in list development to increase its adequacy for application" is unclear; please explain or rephrase.

Methods and Materials

- P4 L57: Explain the meaning of "formal" in "used a properly described formal methodology."
- P5 L8: The abstract states the search was "on 5 May 2025," while here it says "on 21 October 2025." Please clarify.

• P5 L9: The search “ambulatory OR preventable” on the WHO Regional Office for Europe website yielded 1280 results; please explain how these were managed.

• P5 L17: The study flow is unclear; were all studies screened at the full-text level?

• P5 L25: It is unclear what data items were collected; please list primary/secondary outcomes or at least refer to Appendix 2.

• P5 L34: Validating the lists with the Solberg and Weissman criteria is not the same as risk of bias assessment. Please move to a different paragraph e.g. data collection if respective criteria are considered as outcome of interests for extraction.

• P5 L35: The criteria from Diamond et al. 2014 are indicators of Delphi study quality; how was risk of bias assessed in studies not using the Delphi method?

• P5 L45: It is unclear how grouping identified ACSC in larger categories helps in the information managment. The approach risks missing the granulation necessary for effective dissemination, clinical and policy-making relevance. Please consider reporting ACSC at the original leve of detail.

• P5 L52: Correct “completed.”

Results

• P5 L58: The numbers of 1196 retrieved articles and 568 deduplicated records do not match those in Figure 1.

• P6 L14: The sentence “The existence of previous bibliographic support…” is not a result and is already mentioned in the methods; please remove repetitions. And complete with the data on the rest of the criteria in the same paragraph.

• P6 L19: “Eight lists used a database of admissions, discharges, or emergency room episodes” is missing references.

• P6 L21: Reference (8) seems incorrect; did you mean (37)?

• P6 L36: External links as an only source of supplementary materials may not be allowed in text; provide results in a PDF format as an appendix?

• P6 L58: “Delphi studies were the only methodological component whose reporting allowed for risk of bias assessment.” Does this mean other publications did not report any methods? Were they scientific primary studies or just list-reports? How can they be included given the eligibility criteria?

• P7 L5: The quality assessment of Delphi process reporting is not equivalent to risk of bias; a mere “statement of the number of rounds” should not be considered “low risk of bias.”

Figure 1

• Please doublecheck the numbers with the result section for consistency.

• Please explain in text which registers were searched with 0 results.

• Please remove lines where (n = 0) for clarity

Table 1

• Please complete with references (numbers) for better understanding of the results section.

• The categorization of methods ("qualitative, quantitative, and mixed methods") is unclear. Only the Delphi method is classified as qualitative, use of databases as quantitative, and other methods as "other than Delphi qualitative and mixed"? The categories are somehow inconsistent with protocol, and the aim for which the methods were used is not described, especially for databases and surveys.

Table 2

• The ACSCs listed are presented at a rather general level, making them difficult to translate into a clinical setting or policymaking due to insufficient detail. A more target-reader-oriented granulation of the ACSCs would be preferred.

Discussion

• Use a consistent layout for all paragraphs, with subtitles between blocks of text, not to the left.

• P7 L13-16: The first sentence "In this study, we systematically identified available ACSC for European populations and methodologically characterized them" and second " We identified lists with a formal methodological reporting covering six European countries (Germany, Latvia, United Kingdom, Portugal, Spain, Ukraine)." seems redundant; please revise.

• P7 L30: "We did not find the use of any database including primary care data, which we believe could prove of benefit to establish referral processes and the health user pathway within the healthcare system." How does it differ from nationwide real-world databases found in six studies mentioned in the result section?

• P7 L47: The limitations mentioned (no access to individual patient data, no aggregated quantitative data, no exploration of unpublished validations) are less relevant compared to the methodological limitations described earlier in the discussion. Please focus on summing up th main limitations in this paragrapth.

• P7 L59–P8 L6: Caution is needed when interpreting literature not due to the low quantity of studies but due to insufficient external validity and generalizability, or maybe even insufficient quality.

• P8 L9-10: The argument that ACSC diagnoses are less relevant for referring primary care physicians is not convincing. These conditions should be known and prioritized by primary care physicians to avoid complications and hospitalizations. The need for timely access to diagnostic tools is a separate issue.

• P8 L26: The paragraph "From a clinical standpoint…" is difficult to follow; please clarify and remove redundancies.

Conclusions

• P8 L38: Remove the word “social” as it is not mentioned elsewhere in the publication. Consider rephrasing to ”public health perspective” mentioned earlier.

• P8 L41: The sentence “Infectious diseases, chronic cardiovascular (diabetes, hypertension), or chronic respiratory diseases were the most consensual conditions across lists” is repeated from the discussion; please remove.

• P8 L42-44: The sentence “Due to both quality and quantity of available evidence, we are aligned with previous literature in considering the usage of ACSC-related performance indicators to assess primary healthcare to be premature” is not supported by the results or the focus of the study.

• Pages 19–22: There are copies of Figures 1–3 processed with Word’s “save changes” function; it is unclear which is the final version. Please remove what should not be in the final publication.

VERSION 1 - AUTHOR RESPONSE

Reviewer: 1
Dr. Ioannis Kokkinakis, University of Lausanne

Comments to the Author:
Thank you for the new revised version of the manuscript.

Although the research question is interesting and important, the manuscript has many systematic and structural errors that need to be adressed (references in the text, stucture of the manuscript, syntax). Almost all parts (introduction, results, discussion, table 2...) need to be edited by the authors in order for the reader to understand the content.

Table 2 could be more systematic and better structured as well as the text in the main manuscript.

Table 2 has been updated to include the link to the full list of terms and their origin (including parent codes, child codes as well as the origin term). Due to the huge size of the table, we opted to place in the main text only those parent terms that were common to at least 50% of primary studies. The reference to the full-size table was missing and is very relevant. We have opted to add a second sub table with the matching ICD codes that would report to conditions that were present in over two thirds of the lists. This is a far-from-ideal compromise we hope will mitigate some concerns also reported by other reviewer. Regarding the full text, it has now been reviewed.

Data on Prospero are outdated and do not reflect the actual stage of the work.

We apologize for this. We have now updated data on PROSPERO to better reflect the current state of the manuscript. We have updated the title, the risk of bias as we have now opted to add JBI tool for qualitative research.

This work should be published, but only after a thorough resturcturing and correrctions from the authors.

We thank you for your recommendation. You have now revised and made corrections throughout the manuscript to enhance its readability and correctness. We hope the manuscript now answers to the expected and desirable quality standards.

Reviewer: 2
Mr. Marek Czajkowski, Örebro University

Comments to the Author:
Thank you for the opportunity to peer-review this interesting study.
We appreciate your kind comment on the degree of interest of the manuscript.

General Comments

This is a rather short publication (6 pages in current formatting, including a 1-page abstract) reporting an ambitious systematic review of the methods and the lists of Ambulatory Care Sensitive Conditions (ACSCs) for European countries. Due to divided focus the narrative is somehow difficult to follow.

We appreciate your comment. We have made clarifications throughout the manuscript to enhance readability and to clarify some less clear sections. We hope the manuscript may now be more adequate.

This is proved by quite ambiguous eligibility criteria: "primary studies that aimed to develop a list of ACSCs for the general population or subpopulations within European countries, and that used a properly described formal methodology." Both aims are interesting, but the synthesis of ACSC lists is a rather unique outcome and naturally deserves to be the main focus of the study. The methods of the included studies can be described and assessed as part of the systematic review process. Whatsmore, despite eligibility criteria requiring "a properly described formal methodology," studies reporting ACSC lists without such methodology were included (see Results, P6 L58), that needs adequate explanation.

We do agree that the focus of the manuscript is the ACSC lists and their synthesis. We have opted to stress the methodological component as there is no standard

approach to list development. We consider the reporting of methodological component as a key eligibility feature. Some ACSC lists are even considered within primary care practice incentives, which reinforces the relevance of having an adequate methodology employed during list development. We want to stress that we did not include studies that did not report any methodology. Those referred in P6L58 are studies that did not have a Delphi component, but they did report some sort of methodology (either qualitative, quantitative or mixed methods), as reported in Table 1. An incorrect in one of the studies has been corrected.

The outcomes of interest in primary studies are missing from the main text and are only listed in Appendix 2 as Collected variables per study, but unclear if pre-defined.

Due to the list of collected variable, we initially felt that referencing to appendix 2 was an easier approach to enhance readability.

The collected variables for the study have been reported *a priori* in the PROSPERO register (please see Data extraction), with some nuances regarding their names in appendix 2, but the content is the same. Upon your suggestion, we agree some information on the collected variables should be reported on the manuscript (we generally describe them and keep the reference for the full list of variables to appendix 2). We have a mistake in which ACSC conditions are listed only within Delphi studies, which is not the case. It now reads as *"Collected variables per study were the ACSC as well as variables related study metadata, study design, and characteristics of studied databases and Delphi pannels. Full set of are variables are reported in appendix 2."*
The outcomes of interest have been defined in the "Main Outcomes" of the "Outcomes to be analysed" of the PROSPERO register (https://www.crd.york.ac.uk/PROSPERO/view/CRD42022349270), which includes the "- Conditions defined as ACSC and respective ontology definition.". Conditions themselves are the focus, and we apologize if that is not clear. Despite this, we are also very interested in reporting the methodological processes of the primary results – so a critical appraisal of the available evidence can be made.

Consequently, the PRISMA checklist has many "Not applicable" answers in the results section, where respective points should be reported for a systematic review.
It is true that PRISMA checklist has many "Not applicable", though we don't believe this to be a consequence of a lack of definition of outcomes.
We unable to provide some of the items of PRISMA mostly because they required some sort of point estimates from primary studies (for instance, for exploring heterogeneity, sensitivity analysis, identification of reporting bias). Despite these difficulties arising mostly from the type of designs of the primary studies, we attempted to group and synthesise the evidence using tabled data (when not possible to compute

everything within paper-range, we opted to create a website for all identified ACSC – ambulatorycare.med.up.pt).

Additional Comments

General

• The formatting of references should be more consistent: references should appear at the end of sentences, not at the beginning of a new one. For example, correct page 4 line 18 “. (14-17).”, line 37 “. (22)”, line 54 “.(26)”, and page 5 line 18 “. (33)” etc.

We have corrected the references’ formatting.

• The use of ACSC in singular and plural (e.g., ACSC/ACSCs lists, “ACSC are …”) and redundancies such as “ACSC conditions” (P6 L52) should be unified.

We appreciate the comment, and we have now replaced unified everything under ACSC, while keeping only “ACSC lists” to differentiate between the lists and the conditions themselves.

• The term “unselected population” is misleading throughout the article; consider using “general population” instead, as used in the study characteristics and the discussion.

Thank you. We have now replaced “unselected population” with “general population” throughout the manuscript.

Abstract

• In general, it is quite long. Consider removing some details not included in ”PRISMA 2020 for Abstracts Checklist ” or rewriting into shorter sentences.

We appreciate your comment. We have now remove some non-essential information and have provided the same meaning with shorter sentences. We hope this meets expectations.

• P3 L36: Correct spelling to “real-world”.

Corrected**.**

• P3 L38: Regarding conclusions: “Evidence on ambulatory care sensitive conditions is heterogeneous and scarce, with considerable risk of bias.” — it is unclear if evidence is truly scarce when 12 studies were found. Risk of bias

was not assessed for all studies and is always considerable; please consider being more precise.

We do understand that the number of studies themselves is not scarce. Despite this, we meant that the evidence is scarce since it derives from different methodologies, most of the countries do not have evidence generated for their populations, and there is, from our perspective, a lack of validation on real-world data to support the evidence generated through consensus panels.

Generated evidence also is not very coherent. As we have stated in the results, 31.2% of conditions were present in at least a third of developed lists but only 7,8% of unique conditions were present in over two thirds of all lists (and they present in Table 2B).

We understand that the sentence may be misleading in its current format, and we have opted to rephrase to: "Evidence on ambulatory care sensitive conditions is heterogeneous, derives from different methodologies and covers six European countries".

We hope you find this now to be improved.

• P3 L36-38: "Most primary studies included lists which are diagnosis-based, aim at national, unselected populations, and include Delphi components to define ACSCs. This may limit their usefulness to referring clinicians." — Please provide more details what is meant by "usefulness to referring clinicians" or remove the sentence from the abstract.

This sentence was in line with our interpretation that generated evidence currently focus on which conditions that can be safely treated in the outpatient setting, but primary care clinicians may have difficulties using it to inform decisions regarding patient referral. Based upon clinical history and physical examination, which often are the only tools at disposal at community healthcare centers, they may not be enough to achieve a diagnosis, and the referral decision will often be based on clinical scenarios, alarm signs and/or need to rule out more serious conditions. These lists do not inform on clinical scenarios, but rather on conditions or diagnosis.

Despite this, this is an interpretation/discussion, rather than a conclusion, so we have opted to rule it out from the abstract.

• P3 L38-39: "Future inclusion of primary care data could be of use to enhance ACSCs lists' usefulness." — Please clarify the origin of this statement, as it was stated that "Use of nationwide real-world databases was limited to six studies." meaning primary care data was included.

Although this has not been made explicit, the databases from the primary studies were mostly nationwide hospital admission databases, with no databases including primary care. We did not change the terminology of "real world" since it is not mandatory to

have primary care data to be deemed as “real world”. Nonetheless, if you feel it would be more appropriate to specify, please let us know.

In line with the correction from the last point, we have removed the reference to usefulness and put the note towards the generation of more evidence. It now reads “We speculate that future inclusion of primary care data could enhance ACSC evidence.”

Introduction

• P4 L19: Remove “(s)” mid sentence and change the color of references (14-17) to black.

Changes have been made. We appreciate the correction.

• P4 L20: Clarify the meaning of “public” in “Despite their public and economic relevance.”

We believe that ACSC lists are of public health relevance, in presumably being able to better inform whether patients with a certain condition could receive safe care. We agree the expression is not clear, so it now reads “Despite their relevance from the public health and economic standpoint, …”. We thank you for your comment.

• P4 L21: Rewrite into shorter sentences and clarify what “they” refers to in “Despite their public and economic relevance, the earliest attempts to analyze avoidable hospitalizations started with Billings et al (18), who analyzed a list of conditions they deemed preventable and concluded that those patients with poor socioeconomic status and uninsured or with basic coverage insurance were susceptible to increased hospitalization rates for ambulatory sensitive conditions.”

We appreciate the comment. By “they”, we meant “the authors”. We have corrected the text. We also have another correction: where it is read “the earliest attempts” should be “one of the earliest attempts”. Solberg and Weissman criteria preceded the Billings work, and that correction has been now made. We apologise for the mistake.

• P4 L29: Explain what is meant by “subpopulations” in “neither across countries nor across different subpopulations.”

The expression “different subpopulations” refer to subsets of the national, general, unselected populations that could have special characteristics that would justify the existence of separate ACSC lists. One example could be the pediatric population, as well as elderly people in nursing homes. We have revised the text – it now reads as “Rooted in the development of these criteria is the idea that ACSC lists are not necessarily universally applicable neither across countries nor across subpopulations with different characteristics.”

• P4 L31: The words "consistently" and "potentially" are somewhat contradictory in "ACSCs are consistently identified as potentially relevant for primary care assessment." Consider rephrasing.

We appreciate the notice. We agree and remove the "potentially" since there are systems in practice assessing primary care performance that includes hospitalizations by ACSC.

• P4 L32: The statement regarding heterogeneity in methodological quality is unclear; provide a reference or move to the discussion.

Removed. The heterogeneity in methodological procedures will be discussed in discussion.

• P4 L34-42: The discussion about the Delphi method and its limitations feels not appropriate for the introduction; move to the discussion.

This discussion on Delphi was added into the introduction in answering to a previous request from a reviewer. We agree with you and have now decided to remove it and add to the discussion, although in a shorter version.

• P4 L43: The speculation in "The potential absence of information regarding employed methodologies for list development or the further validation of lists used by health authorities for performance assessments yields reasonable questions regarding the usefulness of performance indicators built upon these lists for primary care evaluation" is not suitable for the introduction.

We agree with you. Initially, we meant to justify the interest in understanding whether ACSC lists that are informing real-world performance indicators are methodologically-sound. Despite our desire, we agree that the present formulation is unsuitable and undesirable for an introduction.

We have opted to reformulate the end of our introduction, and have now the following formulation:

"We need to better understand current evidence on ACSC, and how it has been generated and validated, particularly, when used in primary care performance assessment or to inform health policy. To the best of our knowledge, evidence synthesis efforts on ACSC and on methodologies employed for the development of such ACSC lists have not been made for European settings. In this study, we aim to systematically identify and characterize the methodological development of ACSC lists developed for populations in European countries."

• P4 L46: The sentence "assess the employed methodologies in list

development to increase its adequacy for application" is unclear; please explain or rephrase.

We meant that assessment methodological quality would help judging whether a certain list could inform policy or performance assessment. Once again, this is not adequate for an introduction. We have rephrased it, alongside the end of our introduction, and have now the following formulation:

"We need to better understand current evidence on ACSC, and how it has been generated and validated, particularly, when used in primary care performance assessment or to inform health policy. To the best of our knowledge, evidence synthesis efforts on ACSC and on methodologies employed for the development of such ACSC lists have not been made for European settings. In this study, we aim to systematically identify and characterize the methodological development of ACSC lists developed for populations in European countries."

Methods and Materials

• P4 L57: Explain the meaning of "formal" in "used a properly described formal methodology."

We meant by formal the "formal" description of a methodology leading to ACSC list. This has been reformulated and now reads "… Only studies that reported the methodology used for ACSC list development were considered".

• P5 L8: The abstract states the search was "on 5 May 2025," while here it says "on 21 October 2025." Please clarify.

This was an error. The most recent date on the methods section is the correct one, this is, 21 October 2025. The abstract has been corrected accordingly.

• P5 L9: The search "ambulatory OR preventable" on the WHO Regional Office for Europe website yielded 1280 results; please explain how these were managed.

We appreciate the valid question. Although the number of results announced in the page is of that magnitude, the number of studies available on that page are less – there are only 10 pages, yielding 100 publications when sorted by relevance (10 per page). There is even a link for google which is includes posts not originating from WHO, so they were not considered. For those 100 publications, we independently screened for title, and, when in doubt, reached for the full text or pdf, if available. The publications related exclusively to vaccination were not considered. We found publications that had already been published and included (for Germany and Portugal), for which methods were the same. We only deemed eligible an extra publication from Latvia https://www.who.int/europe/publications/i/item/WHO-EURO-

2015-3222-42981-60062, for which there was no included study, but they reported on the methodology for list development.

• P5 L17: The study flow is unclear; were all studies screened at the full-text level?

We appreciate the question. No, we have conducted a two-phase screening, a first phase based on title and abstract evaluations (made independently by two reviewers), and a second phase for which full text were accessed.

• P5 L25: It is unclear what data items were collected; please list primary/secondary outcomes or at least refer to Appendix 2.

We have clarified and improved reporting in the *Data Collection process* section. It now reads "*Collected variables per study were listed ACSC as well as variables related study metadata, study design, and characteristics of studied databases and Delphi panels. We also collected data on the presence of Solberg and Weissman criteria. Full set of are variables are reported in appendix 2.*"

• P5 L34: Validating the lists with the Solberg and Weissman criteria is not the same as risk of bias assessment. Please move to a different paragraph e.g. data collection if respective criteria are considered as outcome of interests for extraction.

We appreciate the correction. It has been removed from risk of bias and added to data collection (and also to data analysis) section of the methods.

• P5 L35: The criteria from Diamond et al. 2014 are indicators of Delphi study quality; how was risk of bias assessed in studies not using the Delphi method?

We appreciate your comment. The risk of bias was not assessed for studies which did not have a Delphi component due to the highly untraditional or hybrid designs. The closest tool we could find that would be eventually JBI tool for qualitative research, which is, in our opinion, a bit to generic and subjective to implement in the remaining articles. https://jbi.global/sites/default/files/2019-05/JBI_Critical_Appraisal-Checklist_for_Qualitative_Research2017_0.pdf. Nonetheless, we are open to evaluate the remaining articles with this, if you feel it would add value (or another tool that would be a better fit).

• P5 L45: It is unclear how grouping identified ACSC in larger categories helps in the information managment. The approach risks missing the granulation

necessary for effective dissemination, clinical and policy-making relevance. Please consider reporting ACSC at the original level of detail.

We acknowledge that the current Table 2 is not ideal. Due to the size of the table containing the original conditions or their mapping ICDs, we opted to create a table corresponding to parent categories that were common to at least half of the conditions. This grouping by parent categories is not ideal and loses important granularity, but serves as a bridge to have a broad view of eventual conditions that are somewhat consensual, as well as to provide the reader with a searchable and interactable tool to go to the highest granularity possible and original descriptions (we have updated the table with link to the full table at ambulatorycare.med.up.pt). We also made it clear now that these are grouping codes. In case you have some suggestion that would simultaneously fit in article-size and keep granularity, we would be happy to receive it and do the according changes.

• P5 L52: Correct "completed."
This has been corrected. We appreciate it.

Results
• P5 L58: The numbers of 1196 retrieved articles and 568 deduplicated records do not match those in Figure 1.

Our apologies for the incorrection. The submitted full text was not adequately updated, only the flowchart. We have now corrected it.

• P6 L14: The sentence "The existence of previous bibliographic support…" is not a result and is already mentioned in the methods; please remove repetitions. And complete with the data on the rest of the criteria in the same paragraph.

We appreciate your comment. We have done so. The new paragraph now includes the results of the other SW criteria and respective references.

• P6 L19: "Eight lists used a database of admissions, discharges, or emergency room episodes" is missing references.

References added.

• P6 L21: Reference (8) seems incorrect; did you mean (37)?

We appreciate the input. Corrected.

• P6 L36: External links as an only source of supplementary materials may not be allowed in text; provide results in a PDF format as an appendix?

We appreciate your concern and would be happy to provide the pdf, but we have three different interactable tables, with one of them generating a pdf of 186 pages long. Due to this, we would rather maintain the website approach, if possible.

• P6 L58: “Delphi studies were the only methodological component whose reporting allowed for risk of bias assessment.” Does this mean other publications did not report any methods? Were they scientific primary studies or just list-reports? How can they be included given the eligibility criteria?

That statement was now removed. We meant that Delphi studies were assessed by Diamond *et al* tool. We have now decided to evaluate the risk of bias for all studies using JBI critical appraisal tools for qualitative research (and updated the protocol accordingly). Although the tool is quite generic, yet it is the best option we have found. That evaluation (Figure3A) is complemented with previously provided evaluations for those with Delphi components. (Figure3B).

The other lists

• P7 L5: The quality assessment of Delphi process reporting is not equivalent to risk of bias; a mere “statement of the number of rounds” should not be considered “low risk of bias.”

We appreciate your comment. In fact, Diamond *et al* assess the quality of the methodological report. We have opted now to assess all studies using JBI critical appraisal tool for qualitative studies, assessing all qualitative components in qualitative and mixed methods studies. We still report the previous usage of Diamond *et al* tool for Delphi studies.

Figure 1

• Please doublecheck the numbers with the result section for consistency.

We have now conducted the doublecheck.

• Please explain in text which registers were searched with 0 results.

There were no registers searched, we kept this in line with the template. We have now removed it.

• Please remove lines where (n = 0) for clarity

We have removed it.

Table 1

• Please complete with references (numbers) for better understanding of the results section.

It has been added.

• The categorization of methods ("qualitative, quantitative, and mixed methods") is unclear. Only the Delphi method is classified as qualitative, use of databases as quantitative, and other methods as "other than Delphi qualitative and mixed"? The categories are somehow inconsistent with protocol, and the aim for which the methods were used is not described, especially for databases and surveys.

We have opted to table the existence of Delphi as a separate category in light of the future application of the Diamond *et al* tool. Nonetheless, we have now evaluated all qualitative components using JBI. We had many "hybrid" designs, so we opted to reformulate the table to have only Yes/No to Quantitative/Qualitative components, as well as the respective correction.

Table 2

• The ACSCs listed are presented at a rather general level, making them difficult to translate into a clinical setting or policymaking due to insufficient detail. A more target-reader-oriented granulation of the ACSCs would be preferred.

We have reported on this on a previous comment.

We acknowledge that the current Table 2 is not ideal. Due to the size of the table containing the original conditions or their mapping ICDs, we opted to create a table corresponding to parent categories that were common to at least half of the conditions. This grouping by parent categories is not ideal and loses important granularity; nonetheless it serves as a bridge to have a broad view of eventual conditions that are somewhat consensual. We have now decided to add a subtable (Table2B) which included the original codes for the conditions common to at least two thirds of the lists.

We also have added a clear indication that the full table is depicted at the faculty website - ambulatorycare.med.up.pt.

Discussion

• Use a consistent layout for all paragraphs, with subtitles between blocks of text, not to the left.

We have now conducted the requested changes, in line with your comment and the editor's comment.

• P7 L13-16: The first sentence “In this study, we systematically identified available ACSC for European populations and methodologically characterized them” and second ” We identified lists with a formal methodological reporting covering six European countries (Germany, Latvia, United Kingdom, Portugal, Spain, Ukraine).” seems redundant; please revise.

We agree with your comment. This now reads “We identified lists covering six European countries (Germany, Latvia, United Kingdom, Portugal, Spain, Ukraine).”.

• P7 L30: “We did not find the use of any database including primary care data, which we believe could prove of benefit to establish referral processes and the health user pathway within the healthcare system.” How does it differ from nationwide real-world databases found in six studies mentioned in the result section?

The nationwide real-world databases were mostly admissions databases for hospital setting. None reported the use of databases that included primary care data, only hospital data. Although they are real-world databases, they mostly like do not incorporate primary care data. Our point is that primary care data could provide an interesting primary care perspective to inform ACSC.

• P7 L47: The limitations mentioned (no access to individual patient data, no aggregated quantitative data, no exploration of unpublished validations) are less relevant compared to the methodological limitations described earlier in the discussion. Please focus on summing up th main limitations in this paragrapth.

Thank you. This part has been reformulated.

• P7 L59–P8 L6: Caution is needed when interpreting literature not due to the low quantity of studies but due to insufficient external validity and generalizability, or maybe even insufficient quality.

We agree with your comment and have updated the discussion accordingly. It now reads “The evidence for European countries has methodological fragilities, with insufficient external validity and generability. This calls for caution when interpreting literature on temporal or regional trends in ACSC.”

• P8 L9-10: The argument that ACSC diagnoses are less relevant for referring primary care physicians is not convincing. These conditions should be known and prioritized by primary care physicians to avoid complications and hospitalizations. The need for timely access to diagnostic tools is a separate issue.

It is true that primary care physicians should know and prioritize these conditions. Nonetheless, we meant that some conditions may not be diagnosis-ready at a primary care setting – and the clinician will have to deal with diagnostic uncertainty and decide on referral without a diagnosis, which may or not be made when the patient arrives at the hospital.

Adding information on “ambulatory care treatable” clinical scenarios rather than “ambulatory care treatable” conditions could further help the referring clinician.

• P8 L26: The paragraph “From a clinical standpoint…” is difficult to follow; please clarify and remove redundancies.

This now reads “From a primary care standpoint, we consider that future research should rather point towards generating and aggregating evidence for primary care referral. This could reduce clinical uncertainty at the ambulatory setting. Improved decision making at the referral decision time could then lead to safer and more efficient shift of certain patients from the hospital to the ambulatory care setting. “

Conclusions

• P8 L38: Remove the word “social” as it is not mentioned elsewhere in the publication. Consider rephrasing to ”public health perspective” mentioned earlier.

This has been corrected. Thank you.

• P8 L41: The sentence “Infectious diseases, chronic cardiovascular (diabetes, hypertension), or chronic respiratory diseases were the most consensual conditions across lists” is repeated from the discussion; please remove.

Removed.

• P8 L42-44: The sentence “Due to both quality and quantity of available evidence, we are aligned with previous literature in considering the usage of ACSC-related performance indicators to assess primary healthcare to be premature” is not supported by the results or the focus of the study.

Removed.

• Pages 19–22: There are copies of Figures 1–3 processed with Word’s “save changes” function; it is unclear which is the final version. Please remove what should not be in the final publication.

Done.

Reviewer: 1
Competing interests: Not applicable

Reviewer: 2
Competing interests: Not applicable.

VERSION 2 - REVIEW

**Reviewer**	**2**
**Name**	**Czajkowski, Marek**
**Affiliation**	**Örebro University, School of Medical Sciences**
**Date**	**31-Jan-2026**
**COI**	

Thank you for your thorough and detailed responses to my comments. I found them informative and helpful, and I have no further comments on those points.

Thank you for correcting errors, accepting many of my suggestions, and rephrasing or removing problematic expressions. These revisions have significantly improved the clarity and quality of the manuscript. I particularly welcome the complete risk of bias assessment of all included studies using validated tools.

I have only a few minor remaining suggestions:

P6 L40 – Please use ” prioritisation ” instead of ” priorisation” for international readers.

P7 L55 – Please insert a space between “ACSC.Reviews”

P8 L 6 – Please use ”generalisability” instead of ”generability”

VERSION 2 - AUTHOR RESPONSE

Reviewer: 2
Mr. Marek Czajkowski, Örebro University

Comments to the Author:
Thank you for your thorough and detailed responses to my comments. I found them informative and helpful, and I have no further comments on those points.
Thank you for correcting errors, accepting many of my suggestions, and rephrasing or removing problematic expressions. These revisions have significantly improved the clarity and quality of the manuscript. I particularly welcome the complete risk of bias assessment of all included studies using validated tools.

We appreciate your kind comment on the improvements conducted as well as your valuable contributions to improve the overall quality of our systematic review.

I have only a few minor remaining suggestions:

P6 L40 – Please use ” prioritisation ” instead of ” priorisation” for international readers. We appreciate it and have now corrected the expression.

P7 L55 – Please insert a space between “ACSC.Reviews”.

We appreciate it and have now corrected the space typo.
P8 L 6 – Please use ”generalisability” instead of ”generability”
We appreciate it and have now corrected the expression.
